# Modeling the Effects of Weather and Climate Change on Malaria Transmission

**DOI:** 10.1289/ehp.0901256

**Published:** 2009-12-07

**Authors:** Paul Edward Parham, Edwin Michael

**Affiliations:** 1 Grantham Institute for Climate Change, Department of Infectious Disease Epidemiology and; 2 Department of Infectious Disease Epidemiology, Imperial College London, London, United Kingdom

**Keywords:** basic reproduction number, climate change, invasion dynamics, malaria transmission, mathematical modeling

## Abstract

**Background:**

In recent years, the impact of climate change on human health has attracted considerable attention; the effects on malaria have been of particular interest because of its disease burden and its transmission sensitivity to environmental conditions.

**Objectives:**

We investigated and illustrated the role that dynamic process-based mathematical models can play in providing strategic insights into the effects of climate change on malaria transmission.

**Methods:**

We evaluated a relatively simple model that permitted valuable and novel insights into the simultaneous effects of rainfall and temperature on mosquito population dynamics, malaria invasion, persistence and local seasonal extinction, and the impact of seasonality on transmission. We illustrated how large-scale climate simulations and infectious disease systems may be modeled and analyzed and how these methods may be applied to predicting changes in the basic reproduction number of malaria across Tanzania.

**Results:**

We found extinction to be more strongly dependent on rainfall than on temperature and identified a temperature window of around 32–33°C where endemic transmission and the rate of spread in disease-free regions is optimized. This window was the same for *Plasmodium falciparum* and *P. vivax*, but mosquito density played a stronger role in driving the rate of malaria spread than did the *Plasmodium* species. The results improved our understanding of how temperature shifts affect the global distribution of at-risk regions, as well as how rapidly malaria outbreaks take off within vulnerable populations.

**Conclusions:**

Disease emergence, extinction, and transmission all depend strongly on climate. Mathematical models offer powerful tools for understanding geographic shifts in incidence as climate changes. Nonlinear dependences of transmission on climate necessitates consideration of both changing climate trends and variability across time scales of interest.

Global climate change remains one of the biggest environmental threats to human welfare over the coming century. Despite representing only one source of possible increases in morbidity and mortality, changes in the severity and global distribution of vector-borne diseases are thought to represent a significant biologic impact of this change [Intergovernmental Panel on Climate Change (IPCC) 2007; Patz et al. 1996]. Along with schistosomiasis and dengue infection, malaria is considered one of the major vector-borne diseases most sensitive to changing environmental conditions (Martens 1998; Martens et al. 1999; Rogers and Randolph 2000), although a considerable range of infectious diseases, including cholera (Pascual et al. 2002), lymphatic filariasis (Sattenspiel 2000), and tick-borne encephalitis (Randolph and Rogers 2000) may also be affected, with potentially profound consequences for human health.

Environmental variables such as temperature, humidity, rainfall, and wind speed affect the incidence of malaria, either through changes in the duration of mosquito and parasite life cycles or influences on human, vector, or parasite behavior (Gubler et al. 2001; Koenraadt et al. 2004). Despite this sensitivity of transmission to changes in environmental variables, and in spite of being one of the biggest causes of worldwide mortality due to infectious diseases (World Health Organization 2008), there is still substantial debate as to the exact role that climate plays in driving malaria epidemics (Hay et al. 2002, 2005; Lindsay and Martens 1998; Pascual et al. 2006, 2008; Patz et al. 2002; Zhou et al. 2004). This uncertainty derives, in part, from the fact that although there is a considerable body of work using empirical-statistical models to investigate the link between environmental variables and transmission intensity of vector-borne diseases (Rogers and Randolph 2000), only limited attempts have been made to incorporate environmental variables into mathematical models describing malaria transmission (Craig et al. 1999; Hoshen and Morse 2004; Lindsay and Birley 1996). The importance of this gap arises from the fact that process-based dynamical models may not only facilitate a greater understanding of the relative importance of internal versus external drivers of transmission, but may better address the effects of complex feedbacks and nonlinear processes typically underlying disease transmission (Martens 1998). Hence, such models, by allowing meaningful capture of the dynamic multiplicative processes and thresholds underlying malaria endemicity and seasonal extinction, as well as disease emergence in new regions as climatic conditions change, provide a credible basis for prediction beyond the range of current climatologic experience. Models also represent valuable strategic tools for policy makers evaluating contingency, mitigation, and abatement strategies and the need for mechanistic models to supplement statistical approaches has been increasingly recognized in recent years (Pascual et al. 2006).

Developing reliable modeling frameworks for integrating the predictions of large-scale climate microsimulations and infectious disease models is inherently challenging, as it involves not only robust treatment of uncertainty, variability, and scale dependency in the modeled physical and biologic processes and input climate data, but also the formulation of realistic climate-based disease transmission models. Modeling work to date incorporating environmental variables into dynamic malaria transmission models has focused almost entirely on the impact of changes in temperature (however, see Hoshen and Morse 2004), despite increasing recognition that temperature and rainfall could have synergistic effects on transmission (Zhou et al. 2004). Moreover, although changes in mean climatic conditions may drive long-term trends in incidence, temporal variability may be epidemiologically more relevant (McKenzie et al. 2001; Zhou et al. 2004), suggesting that understanding the role of climatic heterogeneities constitutes another key modeling requirement. Ultimately, the next generation of process-driven models must also address the changing social and physical environment of at-risk communities, which interact with environmental variables to generate conditions suitable for pathogen establishment, transmission, and extinction.

In this study, we describe the first phase of research in developing an integrated modeling framework for evaluating and predicting the likely impact of climate change on malaria transmission. Although the framework will ultimately seek to combine climate modeling with mathematical models, as well as the socioecology and policy dimensions of disease transmission, we focus on the construction of a generalized, realistic, climate-based malaria transmission model that allows capture of the simultaneous effects of rainfall and temperature on infection dynamics. We follow this up by highlighting how analyses of such models can enable examination of critical and general dynamical issues not addressed to date, namely, the impact on mosquito populations of changes in climatic conditions, analysis of malaria invasion dynamics in disease-free regions and the effects of seasonal variability in climate variables on endemic prevalence, invasion, and extinction. We also highlight the limitations of current mathematical modeling that address these issues, with the objective of stimulating further interdisciplinary research to improve our understanding. We apply model predictions to expected changes in transmission for the Republic of Tanzania as part of an attempt, in partnership with colleagues from the National Institute for Medical Research, to understand the likely impact of climate change on malaria in currently endemic regions and to illustrate the strategic value to policy makers of robust, reliable, and validated mathematical models.

## Materials and Methods

### Climate-driven mathematical models of malaria transmission dynamics

Mathematical frameworks for modeling the effects of climate on infectious disease dynamics may consider deterministic or stochastic transmission models embedded within static or fluctuating environments. The equations governing weather and climate models are deterministic, but there is extreme sensitivity to initial conditions; Grenfell et al. (1995) also documented chaotic behavior in infectious disease models. Thus, the resulting interactions are highly complex, and developing mathematical frameworks to understand the interaction between these systems is inherently challenging. We were motivated to develop and analyze the foregoing model for two reasons. First, there is undoubtedly value in taking a hierarchical approach beginning with generalized models that, although coarse-grained, allow general insights regarding the impact of climate change on malaria transmission. Second, our focus is on unraveling the impact of global change on transmission dynamics, rather than on equilibrium outcomes alone. We believe that understanding such dynamics will, by providing information on disease extinction, emergence, and invasion rates, be of major strategic value to policy makers in developing interventions relevant to health planning.

Our model assumes the standard compartmental structure typical of microparasitic infections in which the mosquito population is divided into the number of those susceptible to infection, mosquitoes exposed but not infectious, and those that are infectious and remain infectious until they die. Humans follow an identical disease history, and we assume permanent immunity such that infectious individuals progress to a removed class (whose individuals cannot become reinfected) at a rate determined by the duration of infectiousness. We also included the effects of weather on *Anopheles* biting rates, demographic parameters, the duration of the *Plasmodium* life cycle on temperature (Martens 1998), and the dependence of the emergence rate of adult mosquitoes on precipitation, which influences the dynamics of immature vectors [see Supplemental Material, (doi:10.1289/ehp.0901256)].

Of course, the effects of climatic variables are far more complex and considerable research is required to quantify and capture such effects within mathematical models. For example, in addition to the recognized effects of temperature and rainfall on the vector and parasite populations under standard or extreme environmental conditions and the interplay between the two (Zhou et al. 2004), disease models should ultimately consider a range of environmental drivers alongside atmospheric variables, including land use changes and hydrologic processes. Here, the biology and epidemiology of transmission, together with the assumed effects of temperature and rainfall, have been deliberately simplified to more clearly illustrate the techniques and insights possible using climate-driven disease models and to strike a balance between simulation models (permitting almost arbitrary heterogeneity) and simpler analytically tractable approaches. Ideally, malaria models should account for a range of more complex epidemiologic factors that affect disease dynamics, such as *a*) more refined modeling of the subtleties of human immunity; *b*) *Plasmodium* strain heterogeneities; *c*) multiple infections and coinfections; *d*) age (and genetic factors) that account for human susceptibilities and infectivities; *e*) human movement patterns; *f*) short-range vector dispersal and heterogeneities in *Anopheles* species; *g*) socioeconomic conditions; and *h*) the emergence of drug resistance.

An important issue in environmentally driven infectious disease systems is identifying the most appropriate spatial and temporal scale at which to model. However, this will be driven by the questions in hand, data availability for parameterization, and the geographic scale over which the model will be calibrated, validated, and applied. Thus, fine-grained approaches will be important to guide interventions at the local level but may not be required for strategic planning on larger scales. Also, they are not only likely to be too computationally intensive to apply over global scales, but may be incompatible with climate data [such as those from General Circulation Models (GCMs)] available to parameterize them. Here, our objective is to adopt a coarse-grained dynamic model to explore the general dynamical impact of climatically driven systems on malaria transmission, with the spatial nature of model output the result of parameterization by local values of temperature and rainfall.

The severity of infectious diseases can be quantified through the basic reproduction number *R*_0_, a static measure of disease severity quantifying the expected number of secondary cases generated per infectious human introduced into an otherwise susceptible population. We can calculate *R*_0_ for the transmission model adopted here by examining the stability of the disease-free equilibrium, providing we ignore temporal variations in temperature and rainfall. In this case, we obtain the standard expression obtained for similar models elsewhere that


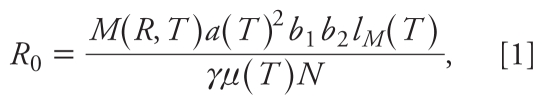


where *M* is the total number of mosquitoes, *T* and *R* denote temperature and rainfall, respectively; *a* is the biting rate per day per mosquito, *b*_1_ is the proportion of bites by susceptible mosquitoes on infectious humans that produce infection, *b*_2_ is the proportion of bites by infectious mosquitoes on susceptible humans that produce infection, *l_M_*(*T*) is the proportion of infected mosquitoes that become infectious, γ is the rate at which infectious humans recover and acquire immunity, μ(*T*) is the daily mortality rate of adult mosquitoes, and *N* is the total number of humans [see Supplemental Material, Table 2 (doi:10.1289/ehp.0901256), for parameter definitions], whereas the derivation of *R*_0_ with temperature and rainfall seasonality is considerably more complex (Bacaër 2007; Bacaër and Ouifki 2007). Estimation of *R*_0_ determines whether malaria outbreaks eventually become endemic (guaranteed in deterministic models when *R*_0_ > 1) and the prevalence to which the system tends, given here by *N*(*R*_0_ − 1) ÷ (*R*_0_ + σ) for the human population [where σ = *a*(*T*)*b*_1_ ÷ μ(*T*) is Macdonald’s index of stability (Macdonald 1957)]. The rate of progression to the endemic state is determined by the invasion dynamics, characterized by the real-time growth rate *r*.

## Results

### Anopheles mosquito population dynamics

The development of realistic, validated models that capture the dynamics of anopheles populations by climate remains an important research area and a crucial component toward improving our understanding of malaria transmission across a range of environmental conditions. For *Anopheles* mosquitoes, the dependence of abundance on climate has been considered within simulation models (Depinay et al. 2004) through empirical work (Lindblade et al. 2000) and via hydrologic impacts on population dynamics (Shaman et al. 2002). Although land–surface models form an integral part of GCMs and topographic and hydrologic factors play an important role in local transmission models, such processes are more readily included within microsimulations that typically lack analytic transparency. Incorporating simpler population models within climate-driven transmission models has received only limited attention to date (Pascual et al. 2006). However, the simplified biology behind such models can incorporate the fundamental mechanisms of temperature-dependent mortality of adult mosquitoes and development characteristics of larvae, which could result in a reasonable model for the rainfall-dependent survival of immature vector stages and a means of capturing the effects of extreme rainfall on existing breeding sites. Moreover, because the effects of seasonality in mosquito abundance enter dynamical models via the transmission term, it is also possible to quantify how much temporal variability is required in the total population size to significantly affect transmission dynamics.

**Figure 1:**
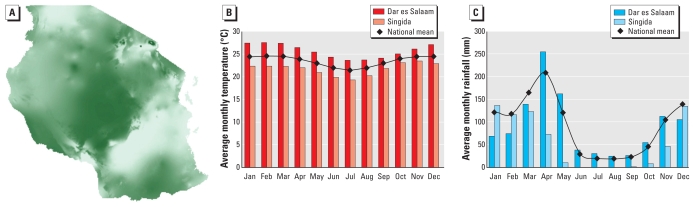
Fadeout probability and monthly temperature and rainfall averages in Tanzania. (*A*) Probability of local seasonal extinction for mosquitoes across Tanzania in April; darker areas indicate a higher probability of fadeout. (*B*) and (*C*) represent average temperature and rainfall values for Dar es Salaam, Singida, and Tanzania as a whole. Data from WorldClim (2009).

Our analysis has provided several novel insights regarding quantifying the impact of climate on *Anopheles* population dynamics. First, although the effect of temperature on vector abundance has a strong physiologic basis and can thus be meaningfully captured by deterministic population models, the effects of rainfall are less predictable and more difficult to quantify. Second, considering the stochastic climate-driven population processes above, we find that the probability of having *M* mosquitoes at time *t* tends to a Poisson distribution with mean λ(*R*,*T*) ÷ *μ*(*T*), independent of the initial conditions, where λ(*R*,*T*) and *μ*(*T*) are the birth and percapita mosquito death rates, respectively [see Supplemental Material (doi:10.1289/ehp.0901256)]. Given this, we can also show that the probability of ultimate population extinction is exp[– λ(*R*,*T*) ÷ *μ*(*T*)]. This expression, together with climate data from WorldClim (Hijmans et al. 2005; WorldClim 2009), allows us to predict mosquito fadeout probabilities over a region, as portrayed in Figure 1A for Tanzania. Given that the transmission model here is deterministic, mosquito fadeout probabilities may be used as a very approximate indicator of malaria fadeout probabilities, but future work should consider a full stochastic transmission model with more robust parameterization for more direct estimations. Figure 1B and C compare the mean temperature and rainfall values for the coastal region of Dar es Salaam and the central region of Singida, highlighting the strong seasonality and spatial heterogeneity in climate across Tanzania, particularly in rainfall. Figure 1A shows how the effects of spatial heterogeneity in the climate drivers of mosquito abundance can be used to determine the likelihood of malaria elimination across an endemic country like Tanzania. Dar es Salaam and other coastal regions generally demonstrate a lower fadeout probability than inland regions, and examination of Figure 1B and C suggest this is more strongly driven by rainfall, rather than temperature, variation between regions. The temporal and spatial heterogeneity in mosquito extinction highlights the strategic importance of assessing control measures driven by regional factors, as well as emphasizing the need to incorporate seasonal climatic variability in transmission models. Environmental drivers other than rainfall and temperature will also drive extinction dynamics, and future transmission models should take a more thorough account of variability in a wider range of atmospheric variables, as well as the potentially strong role of factors such as land use, soil type, local topography, and ongoing vector interventions.

### Malaria invasion dynamics

**Figure 2:**
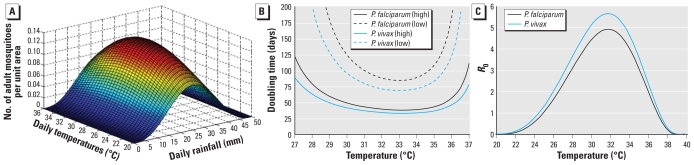
Effect of temperature and rainfall on mosquito population and *Plasmodium* species dynamics. (*A*) The mean number of mosquitoes per unit area as a function of temperature and rainfall. (*B*) Estimated doubling times of *P. falciparum* and *P. vivax;* high and low refer to vector density values: the number of mosquitoes per humans (*M* ÷ *N*). (*C*) The dependence of *R*_0_ on temperature for *P. falciparum* and *P. vivax*.

An important limitation of statistical models is the inability to understand the dynamical properties of disease outbreaks, such as the rate of spread in naïve populations under changing environmental conditions. Here, we consider the transmission model during the early stages of an outbreak when the human and mosquito populations are almost entirely susceptible. We can then apply analytic techniques [see Supplemental Material (doi:10.1289/ehp.0901256)] to derive an equation for the growth rate *r* of the epidemic, and substituting from Supplemental Material, Tables 1 and 3 and *R*_0_(*R*,*T*) from Equation 1 allows numerical solution. We assume sufficient rainfall for *R*_0_ > 1 and consider the outbreak doubling time ln(2) ÷ *r* as a function of temperature in Figure 2A.

We identified a clear temperature window within which the rate of malaria spread is significantly increased (around 32–33°C for both *Plasmodium falciparum* and *Plasmodium vivax*), with the number of cases doubling roughly every month and a half when mosquito density is typical of the rainy season and two and a half months when the vector density drops to half this value. At small deviations from these temperatures, stability of doubling time estimates depends strongly on mosquito density. The shape of the doubling time curves indicates that on either side of this window, fewer mosquitoes survive long enough for the *Plasmodium* parasite to complete its life cycle within the host, essentially because of the rapid decline in vector survival probability at higher temperatures and a rapid increase in the duration of the cycle at lower temperatures. At fixed vector densities, *P. falciparum* and *P. vivax* differ by a few weeks in their rate of spread, with *P. vivax* spreading more rapidly. Figure 2B demonstrates that the transmission rate depends more strongly on vector density than on parasite species, with a doubling in vector density more than halving the doubling time and on the order of months, rather than weeks. This is true of both *P. falciparum* and *P. vivax*.

### R_0_ under static environmental conditions

Under the common, but highly simplified, assumption of vector abundance independent of environmental conditions, we can substitute factors in Equation 1 with parameters in Supplemental Material Table 1 (doi:10.1289/ehp.0901256) and the expressions from Martens (1998) to obtain a temperature-dependent expression for *R*_0_ as


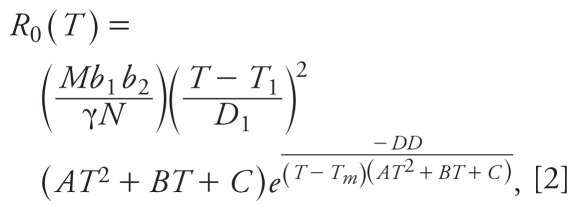


but we have already found that mosquito abundance is strongly dependent on environmental variables. Thus, relaxing the assumption of a fixed vector population size, substituting the dependence on temperature and rainfall from the stochastic population model, and making the improved approximation that the vector population is at a climatically determined equilibrium, we can substitute λ(*R*,*T*)/*μ*(*T*) for the vector population into Equation 2 to obtain a more accurate dependence of *R*_0_ on temperature and rainfall. At this equilibrium, *R*_0_∞*M∞*λ, and hence the dependence of *R*_0_ on rainfall, is the same as the dependence of λ on rainfall. Figure 2A qualitatively illustrates the dependence of *R*_0_ on rainfall, and the dependence on temperature is plotted in Figure 2C. As in the invasion dynamics, the *R*_0_(*T*) curve illustrates an optimum transmission window around 32–33°C [where *T* = *T_max_* (maximum temperature)], and the center of this window is identical for *P. falciparum* and *P. vivax*. It is also easy to see that *R*_0_(*T*) is strongly driven by the probability that mosquitoes survive long enough for parasites to complete their life cycle. Figure 2C begins to address the issue of how transmission may shift with climate change. For a Δ*T* rise in mean temperature, endemic regions where *T* + Δ*T* < *T_max_* will experience a considerable increase in prevalence as conditions become more favorable for transmission, whereas in regions where *T > T_max_*, the survival probability of mosquitoes declines and transmission is reduced. Thus, the major impact of these results is that although the global distribution of malaria will change as climatic variables change, the impact will not always be for the worse. However, subject to sufficient mosquitoes to drive transmission, it is clear that increasing temperatures always increase the probability of emergence in regions where there is currently insufficient transmission to drive endemnicity, as these regions always have *T* + Δ*T* < *T_max_*. Indeed, more robust model parameterization and validation will permit identification of areas where a given shift in average temperatures will permit emergence at doubling rates captured by Figure 2B, as well as a better understanding of how changes in rainfall affect the possibility of emergence and endemnicity.

### The effects of seasonality in rainfall

So far, we have considered a deterministic transmission model within an unchanging environment. Although this yields useful insights, there is generally considerable environmental variability and uncertainty within the system. This may arise from natural temporal fluctuations in environmental variables, parameter inference, or estimation of parameters from other sources (e.g., predictions from GCMs for different emission scenarios). Full consideration of the implications of uncertainty and variability is beyond the scope of this paper; thus, we make only preliminary comments here.

The inclusion of periodic forcing in epidemic models has received attention across a range of infectious diseases (Grassly and Fraser 2006), although the forcing of malaria transmission by climate variability has received only limited attention to date (McKenzie et al. 2001). Recent theoretical advances have considered how seasonality in transmission rates affect *R*_0_ and the growth rate of an outbreak (Bacaër and Ouifki 2007). Understanding the effects of temperature variability is challenging, as temperature dependence appears in multiple places in the transmission model, whereas a preliminary understanding of the impact of rainfall variability is simpler by virtue of appearing only through the vector abundance. Thus, we consider here only the effect of rainfall variability on *R*_0_ and assume no variability in temperature.

Given the strong dependence of vector abundance on rainfall, we make the simplifying assumption that *M*(*t*)∞*R* (*t*)∞[1 + ɛcos(ω*t*-φ)], where ɛ is the amplitude of a constant representing seasonal variation (dimensionless) and ω represents the frequency of seasonal variation (in months^−1^). If *m_max_* represents the maximum number of mosquitoes per human and we simplify the analysis by ignoring the human and vector latent periods, a calculation identical to that in Bacaër (2007) shows that *R*_0_ may be approximated as


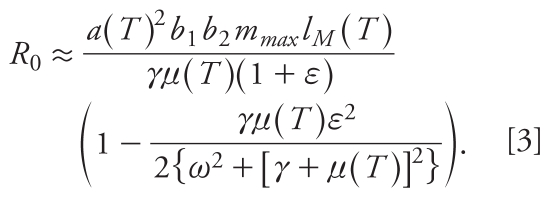


Fitting to historical WorldClim rainfall data for Tanzania and letting *m_max_* = 40, representing typical vector abundance per human during the rainy season, gives ω *≈* 0.65 months^−1^ and ɛ *≈* 0.98. Supplemental Material, Figure 1 (doi:10.1289/ehp.0901256), shows that rainfall seasonality always decreases *R*_0_ in a static environment, with a small amount of seasonality having a more significant impact on transmission around the optimum temperature window of 32–33°C. Given the relationship between *R*_0_ and the level of control required for elimination, this highlights the importance of accounting for uncertainty when using models to inform policy. Here, the effect of not doing so would overestimate the level of resources required for elimination.

### Changes in R_0_ distribution

We predicted and mapped changes in the *R*_0_ distribution of malaria across Tanzania with predicted changes in temperature and rainfall. We use predictions of the HadCM3 GCM for the A2a and B2a emission scenarios from the WorldClim database (Hijmans et al. 2005; Nakicenovic et al. 2000; WorldClim 2009) and apply Equation 3 to compute the value of *R*_0_ per pixel, fitting rainfall data for each scenario to estimate ɛ and ω. We calculate *R*_0_ for the current conditions and values for 2080 under A2a and B2a, computing the *R*_0_ difference map to illustrate the change in *P. falciparum* for the peak rainy season in Tanzania, and we can readily relate changes in *R*_0_ to changes in endemic prevalence when *R*_0_ > 1.

**Figure 3:**
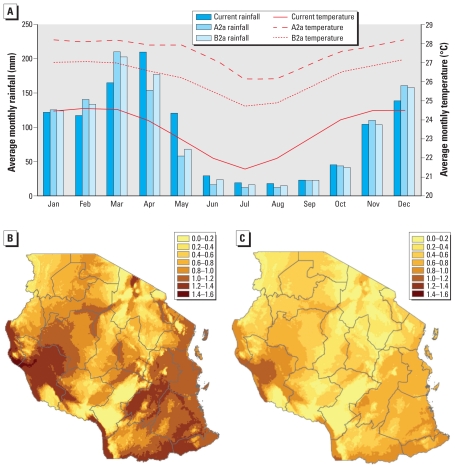
Rainfall and temperature profiles and predicted *R*_0_ changes in Tanzania. (*A*) Current rainfall and temperature profiles for Tanzania versus the predictions of HadCM3 for 2080 under A2a and B2a emission scenarios (data from WorldClim 2009). Predicted changes in *R*_0_ across Tanzania in 2080 under (*B*) A2a and (*C*) B2a emission scenarios where ɛ *≈* 0.98 and ω *≈* 0.65 at present and ɛ *≈* 0.99 and ω *≈* 0.65 under A2a and B2a.

Figure 3A compares the current temperature and rainfall profile for Tanzania with the HadCM3 predictions (averaged across all regions) for 2080. The A2a scenario corresponds to considerable increases in average temperature across Tanzania, ranging from 3.4°C in November to 5.2°C in June, whereas B2a increases (by virtue of being more ecologically friendly) are around 1–1.5°C lower than A2a throughout the year. Figures 3A and B plot predicted changes in *R*_0_ for April (corresponding to the approximate peak in rainfall) for each scenario. As current temperatures are below the peak transmission window around 32–33°C (the current maximum across the country is around 28.5°C), both A2a and B2a predict increases in *R*_0_ across Tanzania without exception. Under A2a, the majority of Tanzania will experience a 4–5°C increase in temperature, and given the current temperature distribution, Figure 3B illustrates that the western regions of Rukwa and Kigoma (particularly the districts bordering the Democratic Republic of Congo), districts within the southern region of Morogoro, the southern borders of Iringa, Ruvuma, and Mtwara with Malawi and Mozambique, and all coastal regions are likely to experience significant increases in *R*_0_ by around 1–1.5 in April. This guarantees endemnicity in these areas and successful invasion of malaria where current climate is not yet suitable for transmission.

Under B2a, most of Tanzania experience a smaller increase in temperature, around 2.5–3.5°C, and although the same regions as before see the largest increases in *R*_0_, these increases are considerably less than under A2a because of the nonlinear dependence of *R*_0_ on temperature. The mean increase in *R*_0_ per region across all regions in Tanzania is 0.87 (variance 0.04) under A2a and 0.52 (variance 0.03) under B2a. It is also worth noting that although both scenarios predict a greater temperature increase in May through to August than in April, rainfall in these months drops off rapidly. Further analysis, based on improved data on the effects of precipitation on mosquito abundance, is required to see if the increase in temperature, potentially moving more regions closer to the peak transmission window (increasing *R*_0_), is offset by the lower vector abundance due to reduced rainfall (decreasing *R*_0_). Such analysis, particularly based on limited data, is beyond the scope of this paper but represents an important area for future research.

## Discussion

The advantages of using process-based transmission models for understanding the impacts of climate change on environmentally sensitive infectious diseases have been widely discussed (Craig et al. 1999; Hoshen and Morse 2004; Lindsay and Birley 1996; Lindsay and Martens 1998). Specifically, mathematical models may overcome problems connected with the use of statistical methods in environmental infectious disease epidemiology via their ability to address multiplicative-exposure effects, nonlinear feedback pathways, spatiotemporal heterogeneities, and complex transmission dynamics. However, it is also clear from our work that significant progress will be made only by developing and analyzing modeling frameworks that are able to realistically capture key linkages between climate and pathogen transmission processes. In this study, we have furthered the progress in this area for climate-based malaria transmission modeling in three key areas. First, in contrast to previous approaches of assuming transmission only when rainfall is greater than certain thresholds (Lindsay and Martens 1998), we, along with complementary research elsewhere (Hoshen and Morse 2004), emphasize the importance of incorporating explicit models of rainfall within dynamic transmission models. Second, we illustrate the importance of modeling the dependence of immature mosquito development on climatic factors, demonstrating how a dynamic stochastic vector population model may be incorporated within transmission modeling frameworks. Finally, although mechanistic models linking climate and malaria have been derived previously, these have been underexploited by virtue of considering only static properties of disease transmission. We show how it is additionally possible to address issues related to infection dynamics, such as emergence and invasion dynamics, the impact of seasonal variability, and disease extinction, all of which have important strategic bearings for assessing the impact of climate changes on transmission within and across regions.

Our analyses of the present model have yielded several new insights regarding the potential impact of climate change on the dynamics of malaria transmission. The first major finding is that by influencing vector abundance, changes in rainfall patterns in particular strongly govern malaria endemicity, invasion, and extinction. In contrast, temperature effects, by affecting in multiple parts of the pathogen life cycle, have a more complex relationship with transmission and a stronger influence on the rate of disease spread, but only when sufficient rainfall exists to sustain vector development and survival. Another key finding, which supports previous suggestions, is that seasonality in rainfall may have marked effects on *R*_0_ and extinction dynamics, even at optimal transmission temperatures. Changing patterns of incidence due to changing environmental conditions on longer time scales, for example, the effects of El Niño-Southern Oscillation, have been considered (Bouma and van der Kaay 1996) and merit further study. Similarly, it is clear that when such studies are combined with concepts from infection dynamics, integrated modeling analyses will prove crucial to improving understanding about how long-term global climate change will affect local environmental conditions and hence whether a region may experience a worsening or improvement in prevalence as global warming increases. To illustrate this approach, we apply these ideas to the generation of risk maps for Tanzania to highlight the sensitivity of spatiotemporal changes in *R*_0_ to predicted shifts in rainfall and temperature under the A2a and B2a scenarios, while taking into account of seasonal fluctuations in rainfall. We show that mosquito density more strongly drives the rate of emergence in nonimmune populations than the infection dynamics of *Plasmodium* species per se, demonstrating how integrated climate- and disease-modeling frameworks allow dissection of spatial heterogeneities in climate-induced malaria transmission and hence the nature of local interventions to mitigate or counter such changes.

Despite advancing new insights into the roles of rainfall and temperature on infection dynamics, our results highlight that considerable work, experimental, theoretical, and policy based, remains to be modeled in an integrated fashion if we are to more realistically capture the impact of climate change on disease transmission. Although certain aspects of transmission dynamics are physiologic (and therefore deterministic) drivers, it is clear that heterogeneities across the human, mosquito, and parasite populations introduce considerable uncertainties into the system. More realistic transmission models need to be spatial if they are to better predict disease persistence and spread, and a key challenge is selecting the most appropriate scale at which to model. This is driven not only by the resolution of available climate data, but also from the knowledge that modeling at too fine a scale may translate poorly into global observables, while oversimplifying local heterogeneities may neglect key processes influencing observations. Another challenge is how best to integrate ecological drivers with sociological processes underpinning disease transmission in vulnerable communities, an area only now beginning to be examined (Lindsay and Birley 1996; Lindsay and Martens 1998; Patz et al. 1996). Improved understanding of the effects of temporal variability on stochastic disease dynamics is also required, and the interaction of these effects with the chaotic nature of climate systems may lead to complex dynamics. The application of complexity theory to climate–disease systems may then be called for to study such interactions (Janssen and Martens 1997). Further experimental research on the effects of atmospheric variables on transmission also requires considerable attention. The survival function of adult mosquitoes with temperature is based here on Martens (1998), assuming a constant humidity of 70–80%, yet we know that the longevity of *Anopheles* mosquitoes is significantly shorter at humidities < 50% (Warrell and Gilles 2002). This illustrates that it is important to understand not only which environmental variables drive transmission, but also how these variables themselves interact.

These issues are all ultimately closely linked with the implementation of intervention strategies for control and elimination. Our results, demonstrating the existence of environmental windows of low transmission, are relevant to the timing and scale of control by representing opportunities for driving out infection reservoirs. A better understanding of transmission forcing by climate from modeling frameworks will also lead to improved insights into issues of local elimination and the potential prospects of global eradication. Questions such as the timing and choice of strategies, the imposition of intervention measures based on local conditions (biological and environmental), and the need to design optimal management approaches accounting for adaptive dynamics (Janssen and Martens 1997) may also be guided by such models. Our finding that mosquito density fluctuations may significantly influence malaria emergence reinforces the need to include vector management options in mitigation or adaptation strategies, highlighting a striking instance of how dynamic climate-driven disease models can usefully guide policy development. In addition to climatic factors, changing prevalence trends may be caused by factors such as changes in surveillance, treatment, resistance, and behavior patterns of vectors and humans, and understanding the relative contributions of these factors is key. Our results show not only how mathematical models are important for highlighting current knowledge limitations and increasing our insights into the underlying processes of climate change and transmission, but also how they are required for facilitating identification of robust strategies for reducing local and global malaria burden.
